# The Rapid Implementation of an Innovative Virtual Diabetes Boot Camp Program: Case Study

**DOI:** 10.2196/32369

**Published:** 2022-01-14

**Authors:** Salim Saiyed, Renu Joshi, Safi Khattab, Shabnam Dhillon

**Affiliations:** 1 University of Pittsburgh Medical Center Harrisburg, PA United States

**Keywords:** telemedicine, diabetes, virtual health, mhealth implementation, virtual diabetes, digital health, mobile health, virtual health, virtual interventions

## Abstract

**Background:**

COVID-19 disrupted health care, causing a decline in the health of patients with chronic diseases and a need to reimagine diabetes care. With the advances in telehealth programs, there is a need to effectively implement programs that meet the needs of patients quickly.

**Objective:**

The aim of this paper was to create a virtual boot camp program for patients with diabetes, in 3 months, from project conception to the enrollment of our first patients. Our goal is to provide practical strategies for rapidly launching an effective virtual program to improve diabetes care.

**Methods:**

A multidisciplinary team of physicians, dieticians, and educators, with support from the telehealth team, created a virtual program for patients with diabetes. The program combined online diabetes data tracking with weekly telehealth visits over a 12-week period.

**Results:**

Over 100 patients have been enrolled in the virtual diabetes boot camp. Preliminary data show an improvement of diabetes in 75% (n=75) of the patients who completed the program. Four principles were identified and developed to reflect the quick design and launch.

**Conclusions:**

The rapid launch of a virtual diabetes program is feasible. A coordinated, team-based, systematic approach will facilitate implementation and sustained adoption across a large multispecialty ambulatory health care organization.

## Introduction

The COVID-19 crisis necessitated an abrupt adjustment to the delivery of outpatient care in order to protect vulnerable populations from avoidable exposure to the virus while providing continuity of care to patients with chronic health issues. One result of the crisis was that telemedicine use exploded worldwide, especially in patients with diabetes [1-6]. In response to COVID-19, UPMC (University of Pittsburgh Medical Center) Central Pennsylvania made an immediate pivot to deliver most ambulatory care via telehealth, resulting in a 1000% surge in telehealth use across all medical and surgical specialties in the early months of the pandemic. In September 2020, we launched a virtual diabetes boot camp program to complement our existing services.

UPMC Central Pennsylvania is a large, integrated, not-for-profit health care system with over 2900 physicians across 7 acute care hospitals and 200 ambulatory care sites, serving over 10 counties in central Pennsylvania. UPMC Central Pennsylvania established their telehealth program in 2013, when a telestroke initiative was launched. The telehealth department has since grown and is led by the chief medical information officer and supported by a director, analysts, clinical implementation consultants, and trainers. It is also supported by a dedicated patient phone and email hotline for telehealth and portal issues. UPMC Central Pennsylvania uses the Epic (Epic Systems Corporation) electronic health record (EHR), the accompanying MyChart patient portal, and the EHR-integrated telehealth platform Vidyo (Vidyo Inc). The platform offers audio and video calls, messaging, file sharing, and automatic vital sign reporting. The diabetes boot camp also uses the website Tidepool (Tidepool Project) [7]. Tidepool is a 501(c)(3) nonprofit organization that provides a web-based platform to coview diabetes-related data entered by the patient.

The all-virtual, holistic diabetes program was conceived and developed in less than 3 months and includes diabetes education, nutrition counseling, coping mechanisms, planned exercise, and medication adjustment via a team of physicians, registered dieticians, and diabetes educators. Patients meet virtually with a team member once a week, alternating between a diabetes educator and a dietician, and regularly interact with the Tidepool application. Follow-up HbA1c (glycated hemoglobin) values are obtained after the completion of the 3-month program.

We started the program at selected pilot clinical sites, refining the program with input from all members of the team before adopting the program at the organizational level. Success was assessed by monitoring HbA1c improvement, weight and blood pressure control, and the number of patients completing the program. By using integrated workflows in the EHR and simplifying the process for both patients and providers, we were able to keep the changes minimal for most of those involved. Educating clinic staff to guide patients through enrollment and the use of the software was critical to the success of the program. Few studies have looked to described generalizable strategies to quickly design, launch, and implement a scalable virtual diabetes program across a large multispecialty ambulatory clinic group.

## Methods

UPMC Central Pennsylvania is comprised of multiple primary care offices consisting of 300 primary care physicians across 50 clinics and 3 endocrinology clinics across a 10-county region. We have a dedicated telehealth department that guides strategy, implements, and supports all virtual programs across the organization. In July 2020, due to the growing need from patients with diabetes to be seen virtually, a diverse group of clinical, operational, and telehealth leaders were engaged to support the launch of a new way of taking care of patients with diabetes who required frequent appointments. The virtual program was developed to alleviate the need for frequent in-person visits during the pandemic using previous and new lessons learned through the pandemic and serve a large geography of patients traveling as far as 1 hour to see their endocrinologist. A quick timeline was decided for the program, and bimonthly meetings were held to optimize the project due to the pandemic emergency and to improve the virtual access of patients with diabetes to doctors.

We were able to embed the new virtual workflows into the Epic ambulatory EHR, facilitating ease of adoption and operational efficiency. A documentation template was created to guide diabetes educators through medication change-related decisions. We also created a unique electronic referral in the EHR for the virtual boot camp to identify it as a new clinical service and for tracking purposes. Both the dieticians and the nutritionist team could receive this referral from physicians. The Tidepool software was installed on provider, educator, and dietician computers. Tidepool provides a diabetes web-based data platform to view data from multiple patient devices and display it together on one timeline. Physicians and staff were trained on using the Tidepool software to download glucose monitoring home data. Our endocrinologists introduced primary care physicians to the program via virtual educational seminars.

Using the endocrinology office as a pilot, a patient registry of patients with diabetes who had laboratory values of HbA1c>8% was generated from the EHR. The endocrinologists discussed the virtual boot camp program with these patients and initiated the referral process for interested patients. Administrative support personnel scheduled the virtual sessions and guided patients through the enrollment process. Patient education, including online instructions for downloading and uploading glucose data (using the Tidepool software), was delivered via the patient portal. If the patients had difficulty following the online instructions, the patient help desk or clinic staff engaged them via phone to guide them through the process.

Each week, the patients met virtually for a 30-minute session, alternating weekly between a diabetes educator and a dietician, to review glucose monitoring data from their mobile app, diet, exercise plans, and lifestyle changes. The virtual meetings were also held to address other education and medication or therapy changes. Progress was followed closely, using real time blood glucose data. Weekly feedback showing improvements helped to keep the patients engaged and adherent to the program. We found that gamification of the process (by tracking the numbers closely and meeting target results) kept the patients motivated throughout the program. At the end of the 12-week program, HbA1c values were rechecked, and each patient had a follow-up telehealth visit with an endocrinologist. Our program’s success was assessed via changes in HbA1c levels, and by patient engagement as measured by the completion of the program. A survey assessed patient satisfaction with the virtual program. Following the launch at the pilot clinic site, the program was opened to patients with diabetes at primary care clinics. Education was provided by a lead endocrinologist champion to primary care clinics about the new virtual boot camp program, the patient criteria, its aim, and the referral process. This study was submitted for Institutional Review Board approval and was found to be exempt since it did not contain any patient-specific data.

**Figure 1:**
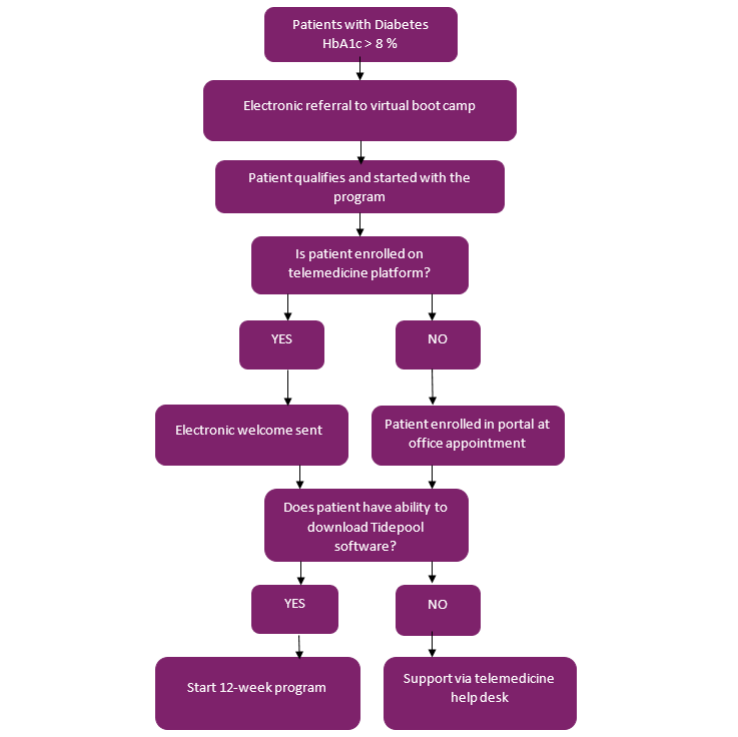
Workflow for virtual boot camp program with a remote enrollment process using video technology. HbA1c: glycated hemoglobin.

Figure 1 describes the workflow for virtual boot camp program with a remote enrollment process using video technology.

## Results

After the program’s launch and successful implementation at the pilot clinic site, it was extended to all 50 primary care clinics in the UPMC Central Pennsylvania network. Within 3 months of the September 2020 launch, we referred and quickly enrolled over 100 patients. Our strategies in 5 broad domains reflect an effort to expedite the launch while creating a sustainable program that can grow beyond the pandemic. Various approaches can be deployed to overcome barriers to implement a virtual program. Using the experience gained through our decades of implementing telehealth programs, we propose recommendations that can be deployed at the system-level for health care organizations to overcome barriers in a rapid time frame (Table 1).

**Table 1:** Implementation strategy and recommendations.

Implementation strategy	Challenges	Recommendations
Project coalition	Need a wide array of representation of stakeholders Agreement on the concept of redesign care Creating a vision to improve care in new models during a pandemic Creating a vision to improve care in new models during a pandemic	Include representation from clinical, operational, and telemedicine area Share vision of need for redesign care Align and remind project goals and scope Create early goals and metrics for the program
Selecting patient population	Technology challenges for patients Is it a right fit for the program? Identify clinical metrics that can be improved with the virtual program Keeping a sustained patient engagement virtually	Need to clearly identify patient population Mock previsit training for patients enrolled in the boot camp Embed virtual boot camp in the electronic health record and workflows Identify compatible platforms and apps Leverage the electronic health record to use metrics to identify and track patients Minimal data entry for patients Provide real time feedback and measure progress with patients
Selecting pilot site	Pilot a clinic that can be model for the organization The practice must be ready to be an early adopter and work with challenges Rapid change can cause disruption in clinic workflow	Identify early a pilot clinic enthusiast of the new program Plan frequent communications and updates as the program evolves Designate program champions that can help adoption
Workflow	Multiple different workflows and platforms can hinder adoption Must be efficient to ease transition Virtual documentation templates can be different	Engage the telemedicine or electronic health record team to keep workflows succinct within the same electronic health record Design patient support and education material Plan remote virtual training for providers and staff Collaborate across disciplines to enhance system changes

Of the 37 patients who completed the program, the mean age was 53 years, with the age range of 22-78 years, 62% (n=23) were female, and 38% (n=14) were male. Moreover, 81% (n=30) were White and 19% (n=7) were African American; 81% (n=30) of the patients had commercial health insurance, and 19% (n=7) were on government health insurance such as Medicaid (Table 2). Of the 30 patients who completed the full program, HbA1c levels decreased from an average of 10.2% to an average of 8.8% (*P*<.001), with a range percentage decrease of 0.7-3%. A survey after completion of the program showed that a majority (n=26, 88%) were very satisfied or satisfied with the virtual boot camp, while 76% (n=23) reported that they felt the virtual boot camp saved them time. A wide majority, 94% (n=28), would recommend the program to their family or friends.

**Table 2:** Demographics of patients who completed the full 12-week virtual program.

Characteristics		Values
Age (years), mean (SD; range)		53.4 (13.9; 22-78)
**Gender, n (%)**		
	Male	14 (38)
	Female	23 (62)
**Race, n (%)**		
	White or Caucasian	30 (81)
	Black or African American	7 (19)

## Discussion

### Study Impact

We demonstrated a quick 3-month implementation of virtual diabetes boot camp with the enrollment of over 100 patients. Our program intervention made an improvement of diabetes in a majority of patients (75%) who completed the program. Our proposed practical steps on guiding strategies can be used for quick implementation solutions for digital health programs. Telehealth has been used in diabetes care since 2000. The IDEATel (Informatics for Diabetes Education and Telemedicine) project, a randomized trial conducted over 5 years in New York, compared usual care with telemedicine among older Medicare beneficiaries [8]. Statistically significant reductions in HbA1c levels, LDL (low-density lipoprotein) cholesterol, and systolic and diastolic blood pressure were seen in the telemedicine group. A meta-analysis of other telediabetes care trials also revealed significant and clinically relevant HbA1C reduction rates (≤-0.5%) [9].

Previous telehealth and diabetes care trials have studied the effectiveness of virtual nurse coaching and mobile health to improve physical activity [10], the impact of self-management skills and psychological aspects in diabetes [11], the effectiveness of virtual care in different genders [12], and web-based dietary interventions [13]. Advances in diabetes telemedicine tools have contributed to a broad availability of solutions; however, barriers to use in terms of acceptance, technical issues, and lack of knowledge remain [14]. A similar study on using telemedicine in treating patients with diabetes, conducted over a 4-month period, showed HbA1c decreased significantly from 9.98% to 8.23% [15]. For patients with diabetes, virtual clinics are shown to reduce treatment burden and to improve therapeutic adherence; it also has societal and psychological benefits that further guide the implementation of such programs [16]. In an mHealth (mobile health) study protocol [17], technologies such as electronic coaching, remote monitoring, and virtual visits showed that patients will improve their activation in diabetes care management, defined as improved self-management.

Our successful holistic interventions for patients with diabetes included a mobile app, diabetes education, nutrition guidelines, lifestyle intervention, therapy adjustment with integrated decision support system, and a dedicated telemedicine help desk phone for support and guidance. Though we faced a few challenges, specifically a quick timeline, we were able to use practical approaches to overcome them (Table 1). Having our primary care doctors and endocrinologist encouraging and enrolling patients likely contributed to the increased adherence and program completion by patients. The relatively short length of the program (12 weeks) and quick results also likely contributed to adherence.

### Barriers and Enablers

Consistent barriers remain such as manual data entry by patients with diabetes, with automation and immediate feedback identified as enablers [18]. The virtual diabetes program offers patients and physicians more time and analytical ability but also offers an alternative to face-to-face visits that may be insufficient [19]. Using integrated workflows in the EHR, we simplified and automated the process for both patients and providers to ease the rapid transition to the new model. Frequent virtual check-ins enabled the patients to remain engaged and provided vital feedback to improve their diabetes. Using a multidisciplinary team, we focused on optimizing the program at the pilot clinic site and utilized the lessons learned for rollout across primary care clinics. Focusing on integrated EHR workflows led to the successful launch of a program in 3 months. The education of clinic staff to guide patients through the enrollment and use of software is critical to the success of the program.

### Conclusion

The virtual diabetes boot camp was launched to improve the overall health of our patients with diabetes and to reduce the need for in-person visits during the pandemic. We successfully launched the program within 3 months, with promising early clinical results and patient satisfaction. The program facilitated frequent engagement between the providers and the patients, decreased the burden for the providers, and increased communication between members of the provider team.

We recommend aligning organizational goals to strategies (Table 1) for the rapid implementation and rollout of a virtual diabetes program. Health systems are finding it challenging to develop effective strategies to address diabetes with the growing shortage of clinicians and health care professionals. Digital strategies such as our virtual boot camp program can help alleviate this burden [20]. The strategy guidelines have been instrumental for our clinic’s rapid transition to telehealth. Our strategies can be adopted by other organizations wishing to launch their own virtual diabetes programs. Most health care organizations have the necessary staff and providers to launch such a program, but only require practical guidelines on technical and operational workflows to deploy it. Though our program’s focus was on patients with diabetes, our strategies could be adapted to manage other chronic diseases virtually. The first step is to form a stakeholder group of leaders who are willing to experiment and launch new ways of delivering care virtually. The key to implementing these strategies is to use the momentum COVID-19 has given to telehealth, sharing the vision of the program with the organization and coordinating the project across different teams.

We launched the program quickly in the endocrinology clinics, but it took sustained educational and communication efforts by primary care clinics to improve adoption across the organization. Sustained effort is needed to successfully roll out a new virtual program and to engage physicians and patients in a multispecialty, large clinic organization. Frequently highlighting the program benefits and continuously monitoring progress is vital to adoption. We were able to use our dedicated patient telehealth help desk to support patients struggling with the telehealth platform. In our experience, a previsit education intervention led to an easier and more successful virtual visit by patients, as confirmed by others [21]. We realize that smaller clinics may not have such a resource and propose training the clinic support staff so they are prepared to help patients. In conclusion, an expedited implementation of virtual programs within large multispecialty US health care systems is possible. In the future, we plan to increase enrollment by enhancing criteria for patients who can be referred to the program; this will lower the threshold for patients who need interventions, such as patients at risk of diabetes or those who are prediabetic. We also plan to include group visits to improve the efficiency of the program. Future studies can look at whether patient interest and engagement will be sustained once temporary pandemic measures are relaxed. We envision we will continue to grow this program as it was set up for a long-term goal beyond the pandemic. The strategies and infrastructure set up will be used to facilitate similar virtual digital health programs across specialties.

## Abbreviations

HbA1c: glycated hemoglobin
EHR: electronic health record
IDEATel: Informatics for Diabetes Education and Telemedicine
LDL: low-density lipoprotein
mHealth: mobile health
UPMC: University of Pittsburgh Medical Center
